# Bacteria Inside Semiconductors as Potential Sensor Elements: Biochip Progress

**DOI:** 10.3390/s140611225

**Published:** 2014-06-24

**Authors:** Vasu R. Sah, Robert E. Baier

**Affiliations:** 1 Harvard-MIT Health Sciences and Technology Division, Cambridge, MA 02139, USA; E-Mail: vsah@partners.org; 2 Industry/University Center for Biosurfaces, State University of New York at Buffalo, Buffalo, NY 14214, USA

**Keywords:** biochips, germanium, crystals, nucleation, *Pseudomonas syzgii*, fabs, optics

## Abstract

It was discovered at the beginning of this Century that living bacteria—and specifically the extremophile *Pseudomonas syzgii*—could be captured inside growing crystals of pure water-corroding semiconductors—specifically germanium—and thereby initiated pursuit of truly functional “biochip-based” biosensors. This observation was first made at the inside ultraviolet-illuminated walls of ultrapure water-flowing semiconductor fabrication facilities (fabs) and has since been, not as perfectly, replicated in simpler flow cell systems for chip manufacture, described here. Recognizing the potential importance of these adducts as optical switches, for example, or probes of metabolic events, the influences of the fabs and their components on the crystal nucleation and growth phenomena now identified are reviewed and discussed with regard to further research needs. For example, optical beams of current photonic circuits can be more easily modulated by integral embedded cells into electrical signals on semiconductors. Such research responds to a recently published Grand Challenge in ceramic science, designing and synthesizing oxide electronics, surfaces, interfaces and nanoscale structures that can be tuned by biological stimuli, to reveal phenomena not otherwise possible with conventional semiconductor electronics. This short review addresses only the fabrication facilities' features at the time of first production of these potential biochips.

## 1. Introduction

**Figure 1.**
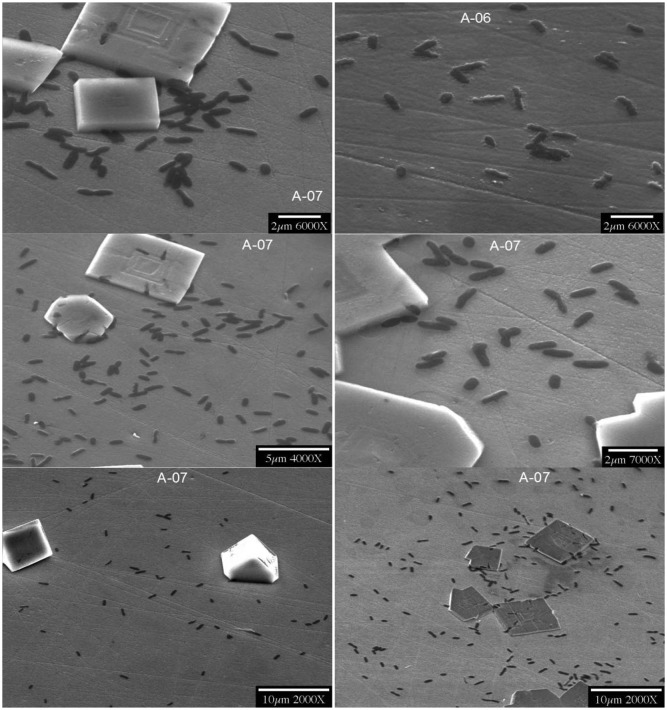
Germania-*Pseudomonas syzgii* compound crystals first observed forming in the University of Arizona fabrication facility in the year 2000.

Although it is well known that bacteria can be trapped within mineral surroundings, it was entirely unanticipated that the amazingly organic-free highly purified water of semiconductor fabrication facilities could uniquely harbor extemophilic microbes that would become trapped within germania crystals that they self-nucleated on germanium sensor plates. This was especially interesting because the same germanium substrata exposed to other flowing water streams readily collected conventional biofilms of organisms and their overlying exopolymeric slimes without such bio-corrosion and nucleation. Thus, in July of 2000, NSF announced this potential breakthrough technology for producing highly selective sensing materials now missing from environmental sensors (NSF Media Tip “Armored Microbes Could Lead to New Biochips”, by Amber Jones). This new technology predicts translation of the scientific concept of “biochips” into actual fabrication of functional devices for the solution of important sensor development problems. Features of ultrapure water (UPW) systems used in semiconductor manufacturing operations (fabs) that may be exploited for creation of true “biochips”, with the possibility of sensitivities and specificities unique to living microbes being functionally integrated into semiconductor crystals, require further exploration. Bacterial contaminants can be found in all conventional water supplies, but are unexpected in the starkly protein-free ultra-purified waters under nitrogen blankets at fabs, especially at sites that are also UV illuminated. In microelectronics manufacturing, device yields are limited by even the smallest bacterial presence and thus justify these fastidious anti-bacterial measures. Described here are the past decade's results in trying to understand and more-simply reproduce this process outside of fabrication facilities, with a review of the then-employed technologies used to guarantee contaminant-free water which appeared exclusively capable of providing the bio-corrosion bacterial entrapment result. This required that one superseded common practices of bacterial detection that relied upon the comparatively crude techniques of visual microscopy and the growth of bacteria on microbiological media. The major analytical deficiency that was overcome was to shift the emphasis from quantifying bacteria that are suspended in the water, to measuring the far greater number that attached to, and were periodically released from, wetted surfaces. From these observations, it is now expected to be possible to have these and similar extremophilic bacteria embedded into semiconductors in ways that will allow those combinations to modulate current flow as effective biotransistors—as well as to eventually use integrated circuit technology to “listen to” bacterial metabolic processes. Figure 1 is an example of the nearly perfect crystals first found within the fabrication facility at the University Arizona, Tucson, Arizona in 2000, and served as a model for more recent attempts to reproduce these findings in the laboratory. The germanium substrata were prism-shaped plates capable of having their surface layers analyzed with a sensitivity of as little as 10 Å to any homogenously spread “conditioning” films of proteins or overlayers of exopolyysaccharides of conventional biofilms, and none were detected. The spectra showed only the new appearance of nucleated germania crystals formed during controlled slow flow of the fab water to waste. It was surprising to see, in the scanning electron microscopy of these plates, the presence of both trapped and adjacent bacteria that later proved to be *Pseudomonas* (now called *Ralstonia*) *syzgii*, an organism of previously unknown ability to thrive in such fastidious environs.

## 2. Experimental Section

In the past decade, numerous lab bench manufacturing methodologies were tried to reproduce this phenomenon, and involved examining the bacterial nucleation and crystallization steps of germanium and silicon by various purities of water, statically and under flow, with simultaneous illumination by ultraviolet energies, concluding that flowing Ultra-Pure Water (UPW), naturally containing or enriched with extremophile *Pseudomonas syzygii*, was the best combination to create the desired bacteria-nucleated crystals on germanium—and, unfortunately, not yet silicon—plates. Scanning Electron Microscopy showed possible nucleation steps and patterns toward routinely fabricating these materials, mainly depending on flow rate (displayed later) and duration of germanium surface interactions. The nucleation events started with square and circular oxide deposits surrounding active attached bacteria, with apparent response to diffusing or spreading metabolic products. Amorphous germania moats were developed around square crystalline growths, with bacteria in ring centers, sometimes in multiples. Further distances of UPW flow along the prisms/plates of germanium showcased amorphous phase dissolution and crystal “ripening”, followed by crystal shedding to downstream crystal clusters [1]. Duration of flow, flow rates and pH changes were recorded, and established as controllable factors for developing these crystals.

Although this work has concentrated on *P. syzgii*, other extremophilic microorganisms might be similarly entrapped within semiconductor crystals. Further work is now required for discriminating between nucleation by any spectrally undetected microbial exudates or peculiar features of microbial surfaces directly, to interrogate the crystals grown with advanced bio-photonic and electronic probes [1].

### 2.1. Methods and Materials

#### 2.1.1. Ultra-Pure Water (UPW)

Ultra-Pure water was stored in a Nalgene polymer container at a temperature of 5 °C. It was obtained from the University of Arizona Center for Micro-Contamination Control unit, where the Figure 1 observations were first made.

#### 2.1.2. Bacterial Culture and Techniques

##### Bacterial Sample

The bacterial strain was identified and extracted from the University of Arizona (UofA) UPW system for semiconductor fabrication [2]. This strain, called MF254A—*Pseuodomonas syzygii*, is obviously an oligotrophic bacterium, that is recoverable by culture from both refrigerator-stored UPW supplies and from frozen stocks (for over ten years, now) and remains viable under these adverse conditions. Originally frozen specimens from UofA were continuously stored frozen and separate 5 mL vials were thawed in a refrigerator and used whenever required.

##### R2A Agar

A low nutrient medium was required for successful re-growth, Difco R2A, which contained 0.5 g yeast extract, 0.5 g Difco Proteose Peptone no. 3, 0.5 g Casamino Acids, 0.5 g Glucose, 0.5 g soluble starch, 0.3 g of K_2_HPO_4_, 0.05 g MgSO_4_·7H_2_O, 0.3 g Sodium Pyruvate and 15 g agar/liter of lab quality water. For preparing plates, 18.2 g of R2A concentrate was diluted in 1 L of water (final pH = 7.2) [1]. The R2A agar was purchased from BD (Becton Dickinson) Difco (Lot 1291882).

##### Sterilization

Autoclave—Glassware and pipettes were sterilized by autoclave, using a temperature of 121 °C at a pressure of 15 psi. The autoclave was a Sterilimatic STME from Market Forge Industries Inc. (Alfa Medical, Westbury, NY, USA).

Radio Frequency Glow Discharge Treatment (RFGDT)—also known as Plasma Cleaner/Sterilizer, from Harrick Scientific Inc. (Ossining NY, USA; now Harrick Plasma Inc., Ithaca NY, USA) was used for glow discharge treatment of the germanium (Ge) prisms. RFGDT used was a room-air plasma with a delivered RF power of 30 W and frequency of 35 MHz.

##### Flow System

The recirculating flow cell system comprised three major parts: (A) flow cell (B) peristaltic pump (C) UPW Container. The Ge prisms were used in the flow cells developed for this specific purpose [3,4]. Each parallel-plate flow cell was a combination of two molded Sylgard half cylinders (Dow Corning, Midland, MI, USA), holding two separated Ge test plates with dimensions 5 × 2 × 0.1 cm. There were also two specially designed Sylgard shims between the two half cylinders to form a uniform flow path between the plates. These created a 0.156 cm gap between the two half cylinders modifying the actual flow path to 5 × 1.3 × 0.156 cm and a total contained static volume of 1.014 cm^3^. At the inlet and outlet, Teflon connectors were inserted which converged with tubing to create the flow path. Two Plexiglass (2.5 mm thickness, polymethylmethacrylate, Evonik Cyro LLC, Parsippany, NJ, USA) were used as shells for securing the Sylgard half cylinders with the help of two stainless steel hose clamps. To eliminate air entrapment, each flow cell was vertically aligned with the flow moving from bottom to top. The tubing used was Masterflex (96400-13; Cole-Parmer, Vernon Hill, IL, USA) with a diameter of 0.03 in (0.8 mm). The total length of tubing used for the flow unit was 75 cm divided into two parts (one from the pump to the flow cell and other from the flow cell to the UPW container) [1]. Two micro-pipette (1 mL) tips were used as connections between the original Teflon connectors and the Masterflex tubing. The process of flowing UPW over germanium surfaces consisted of recirculating the UPW through laminar-flow loops developed across the 50 × 20 × 1 mm germanium (Ge) prisms inside the described flow cells, followed by microscopic and spectroscopic examination of the surface deposits.

##### SEM (Scanning Electron Microscopy)/EDS

For SEM imaging, a S4000 SEM Hitachi (Tokyo, Japan) was used. Images were recorded using an acceleration voltage of only 5 KV, and no conductive over-coating. Samples were prepared by flowing UPW over germanium prisms (50 mm × 20 mm × 1 mm) using the described flow cell system and letting the prisms (plates) dry under normal room temperature in a sealed Petri dish. EDS (Energy Dispersive X-Ray Spectroscopy), using an inbuilt device within the SEM S4000, used emitted X-rays for elemental analysis.

##### MAIR-IR (Multiple Attenuated Internal Reflection—Infra-Red Spectroscopy)

MAIR-IR spectra were recorded using a Perkin Elmer (Shelton, CT, USA) Infra-Red Spectrophotometer assembly. A mirror device was the additional accessory used with the sample holder. The accessory consisted of a base with three angle positions (30, 45, 60 degrees) and different mirrors (two spherical and one flat) along with the holder assembly. The setting of 45 degrees was used with 45-degree entry-angle trapezoidal germanium prisms.

##### XPS (X-Ray Photoelectron Spectroscopy)

The XPS instrument (also called ESCA—Electron Spectroscopy for Chemical Analysis) used in these investigations was a SSI Model SSX-100 (Surface Science Instruments, Pleasanton, CA, USA). It had a monochromatic Al X-ray source using a spot size of 400 micrometers. It used soft x-rays (with a photon energy of 200–2000 eV) to examine the specimens. Processing and component fitting of high resolution electron energy spectra were performed with Casa-XPS software.

## 3. Results and Discussion

**Figure 2.**
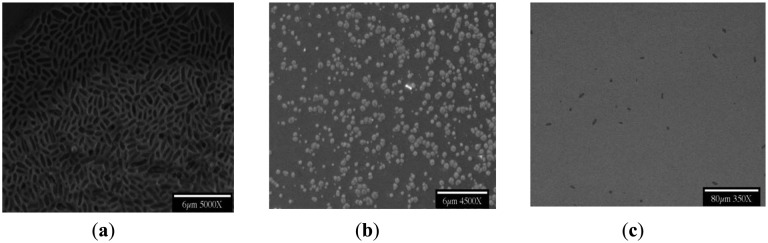
SEM images—From Left to Right: (**a**) Cultured *Pseudomonas syzygii* using R2A Media (Difco) growing in high quantity; No flow UltraPure Water (UPW) (**b**) Dried UPW on a Germanium (Ge) prism enclosed inside a Petri dish; (**c**) Single bacteria seen at various locations of the Ge surface (identical to bacteria in Arizona FAB).

Figure 1 has shown typical scanning electron microscopic (SEM) views of the Year-2000 discovery inside flow cells, as taken directly from the fab at University of Arizona Micro-Contamination Center, presumably grown by nucleation upon microorganisms from ultrapure water-dissolved semiconductor substrata. Although attempts were made to optimize the flow rate, duration, pH and temperature required for fabricating these crystals at the bench scale, these efforts have only partially succeeded to date. A brief summary of the flow rates, pH conditions, and durations tested is provided in Tables 1, 2, and 3, and the findings are illustrated in the following Figures. The frozen lab-utilized *P. syzygii* cultures, originally obtained from University of Arizona, were successfully grown to abundance on R2A minimal nutrient media (Figure 2) and were found by microscopy to be essentially identical to the microbes in stored UPW from the same facility at Arizona.

Bacteria first attached to the RFGDT Ge surfaces in the form of a single bacterium or a small bacterial colony. The following equation describes the apparent basic reaction observed at the UPW/Ge surface: Ge + H_2_O → GeO_2_ + 2H_2_. The event of UPW flow over the surface of Ge first led to partial dissolution/corrosion of the germanium followed by crystal nucleation and saturation, as described below.

After tedious testing, the optimum flow rate and exposure time were 1 mL/minute (3.2 s^−1^ shear rate ) for 4 days at room temperature, resulting in densest crystal arrays at the prism central zone (2–3 cm from inlets). A different nucleation mechanism and saturation of crystal formation was apparently induced, at different flow rates and exposure times, especially with a higher shear [1]. The flow rates and durations of the ultrapure water's interaction with Ge surfaces were thus seen to be important factors in the growth patterns of these oxide crystals.

**Figure 3.**
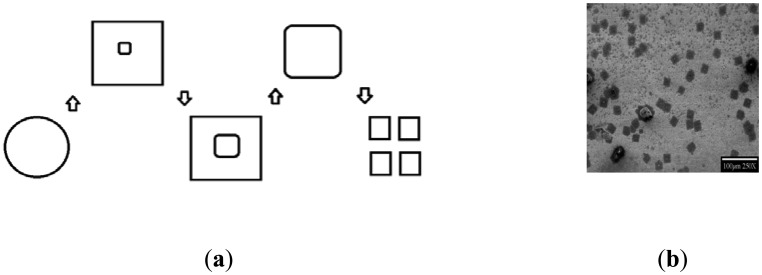
Flowchart representing nucleation, growth, and separation of bio-crystals.

When bacterial nucleation of such crystals was examined, *P. syzygii* species (extremophilic and oligotrophic) in ultrapure water possessed a unique nucleation pathway (Figure 3).

**Figure 4.**
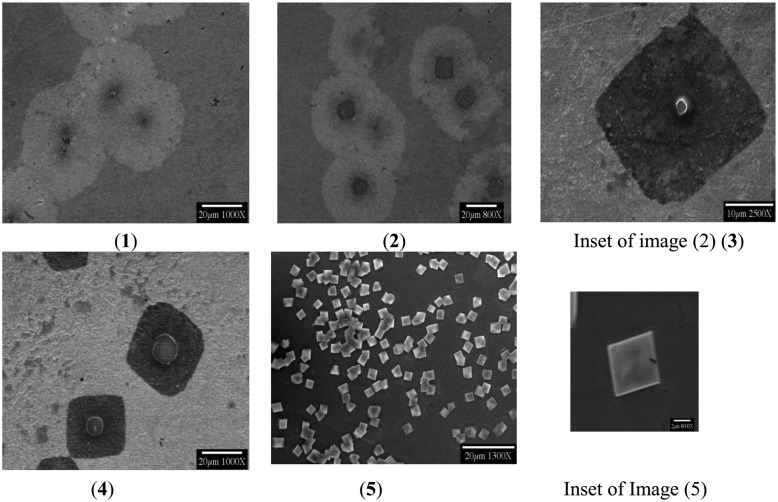
(As observed on the Ge prism)—From (1) Seeding due to the central bacterium in circular deposits (2) Growth of oxide-embedded seeds with bacteria (3) Crystal growth initiation after formation of square oxide moats (3b) Vast number of square oxide moats seen (4) Crystal formation in the center, shape depending upon the deposit morphology. (5) Formation of 3–5 micrometer bacterial crystals—Germanium Oxide crystals, with bacteria in majority of them (Inset).

These events began, in the test systems so far evaluated, with square and circular oxide deposits centered at the active bacteria, apparently due to the spreading of IR−undetected metabolic products (Figure 4). The bacterial exudates germinated into amorphous germania seeds for square crystalline growths and these later grew into different shapes. The bacterium was usually retained in the crystalline center, with few exceptions. The secreted metabolites evident in SEM images of formed circular and square germanium oxide deposits always showed bacteria in the centers.

An edge-moat formation was also observed, where nucleation first occurred at the extreme of cellular and square exudate zones. Crystal dissolution and “ripening” at different flow rates followed this general pattern observed with a rate of 1 mL/min for a period of 4 days. Usually, a single bacterium formed a circular hillock around it, probably with its secretory materials, serving as a “seed” crystal for crystal formation with a square boundary around the bacterium, with continuing growth leading to a forming larger crystal which emerged from the existing one.

The crystals which were slowest in growth converted to larger structures, fragmented to a certain extent and saturated the interfacial fluid to form additional homogenous crystals downstream. A short and speculative description of these events of nucleation and crystal growth, as observed, offers the explanation that Ge oxidation to GeO_2_ occurs from UV—dissociated water corroding the Ge surface while releasing protons, which keeps the organisms alive by releasing protons and driving the metabolic processes [1].

Even more speculative has been discussion that the process of seeding and nucleation of GeO_2_ crystals may preferentially occur at the grain boundaries and dislocations of the polycrystalline semiconductor crystals. The etching and corrosion produces the holes and electrons at the surface of semiconductors; corrosion being faster at the defect. Semiconductor dissolution takes place at the anodic sites and the oxidation occurs at cathodic sites. In the absence of an oxidizing agent (like Oxygen) and metal ions dissolved in UPW, the discharge of hydrogen ions occurs at a very slow rate and becomes the main path to corrosion. Protons tend to disrupt chemical bonds and shift the valence band to the forbidden energy region. Negatively charged bacterial cell walls could cause increased hole density (making it a P Type Semiconductor), increasing the conductance. These holes, produced by biological impurities, can be amplified by the current passing through the crystal.

**Figure 5.**
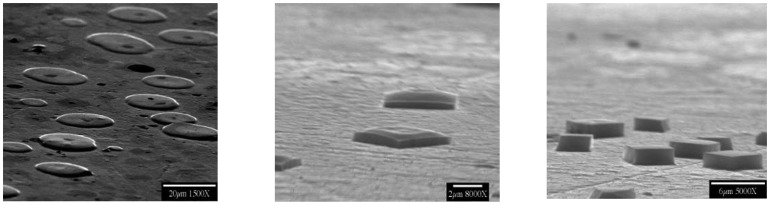
(Inclined SEM—at 75 Degrees): From left to right, showing an inclined SEM nucleation of the bacterial seeding at the center of the deposit, eventually forming bio-crystals.

Low voltage SEM imaging was performed where the beam energy was only 5 KeV, to achieve a more detailed surface morphology image. Conventional accelerating voltages near 25 KeV did not yield desired images. The SEM images displayed brighter areas where the retained electrons were most dense and black ones, showing suppressed electron emission from the substratum when bacteria were attached. Bacterium division was often evident in SEM images, indicating the in-process replication and growth of this species. Inclined SEM, at 75 degrees, helped identify the heights of the chips (about 700–800 nm) and bonding with the parent surface. Some chips remained fixed and others were seen to be detaching from the surface, showcasing a clear rectangular outline from the cross-sectional views (Figure 5).

**Figure 6.**
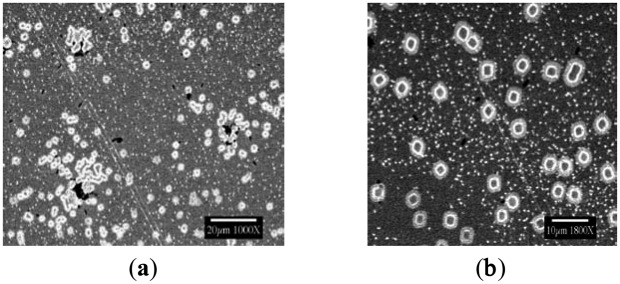
Exposures for more than 4 days showed corrosion crystals without bacterial entrapment.

The shifting nucleation pattern (Figure 6), observed at a flow rate of 1 mL/min and longer duration of 7 days, included the formation of the crystals from agglomerated bacteria/small colonies, corroding the germanium surface and forming similar crystals as observed in the above condition. The crystals grown late were observed to be relatively free of included bacteria.

**Figure 7.**
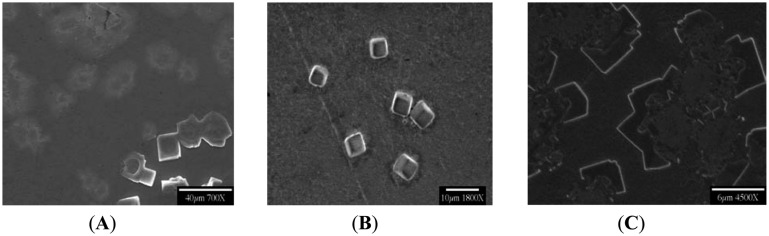
SEM images (**A**) pH = 5.5 at flow rate of 1 mL/min; Normal UPW pH = 7 (**B**) flow rate of 1 mL/min (**C**) flow rate of 2.5 mL/min and more.

**Figure 8.**
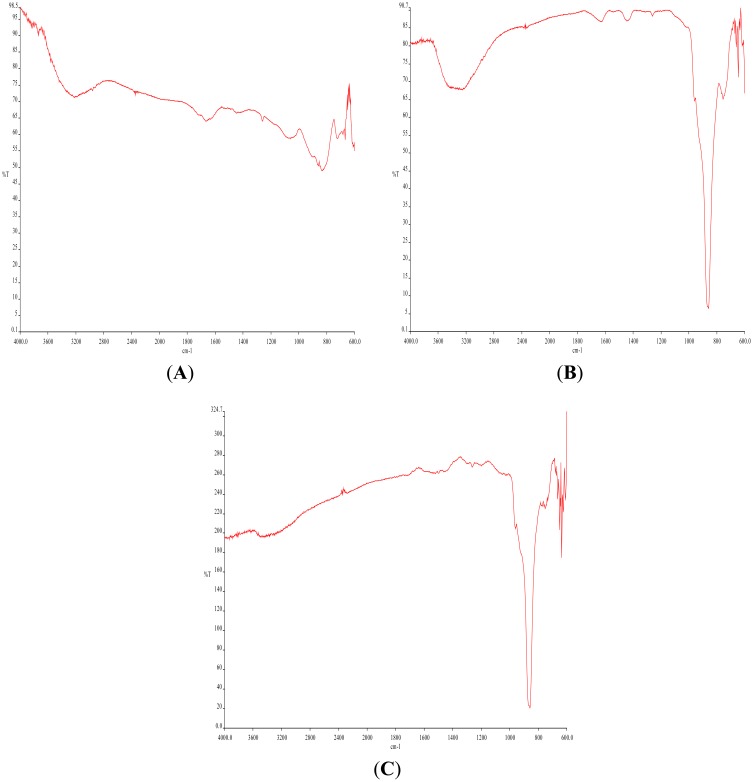
Following the same pattern as in Figure 7—MAIR-IR Spectra—A, B, C.

At varying flow rates, with deliberate changes in pH, even more variance has been shown (Figures 7 and 8). MAIR-IR spectra and SEM images clearly depicted these changes in morphology and material composition. They remain to be assessed, along with fab features described below, as important factors in the desired routine biochip manufacturing process.

MAIR-IR (Figure 8A) of the stored UPW had no peaks specific to any contamination. Figure 8A and C shows MAIR-IR Spectra with comparatively less –OH % transmittance (3200 cm^−1^), less Amide I/II (1550 and 1650 cm^−1^) of the bacterial proteins, and Ge oxide (850 cm^−1^) peak; as compared to (Figure 8B). The band at 1450 cm^−1^ (Figures7B and 8B), the carboxyl band from the exopolymeric carboxylate bacterial metabolites zone excreted, was often abundant. It was never observed, however, as a precursor to the appearance of the corrosion-produced crystals, a considerable surprise given the most common path of biofilm formation: “conditioning” protein film, bacterial attachment, exopolymer secretion. The pH of UPW played a major role in forming the observed crystals. When the pH was changed to 5.5 (originally being 7), this change in the H^+^ to the acidic conditions dissolved pre-existing crystals (Figure 8A), leaving negligible GeO peaks. The prolonged duration of flow with the higher flow rates of 2.5 mL/min and 4 mL/min led to secondary crystallization, leading to overgrowth of the crystals and agglomeration (Figure 7C). Flow rate of 1 mL/min EDS results depicted the following general composition—germanium as 46%, oxygen as 45% and carbon as 9% by point scanning at the centers of the bio-based crystals.

**Figure 9.**
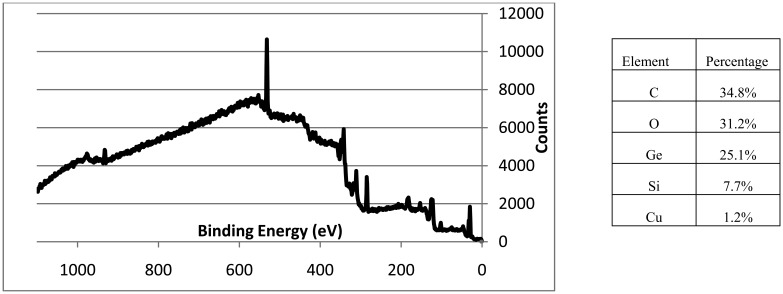
XPS Spectrum showing the formation of a specific oxide of Ge—GeO_2_ with Carbon and trace amounts of Si (from the flow cell)—(C1s at 284.6 eV for C-C, 286.4 eV for C-O, 288.6 eV for O=C-O; O1s peak at 531.9 eV for GeO_2_; Ge3d at 32.7 eV for GeO_2_; Si2p at 102.2 eV).

A small MAIR-IR methyl-silicon peak (1250 cm^−1^) in Figure 7B was confirmed by XPS results (Figure 9), to be contamination from shims of the flow cells. Trace amounts of copper (1.2%) from the instrument were also seen and noted. XPS spectra (Figure 9) confirmed the major presence of GeO_2_ and carbon with C-C, C-O and O=C-O linkages.

It is not likely that all microorganisms will be amenable to survival in similar processes, or to incorporation in secondarily nucleated crystals. Process chemicals such as ionic and non-ionic surfactants can be used to improve the wettability, reduce the roughness, and reduce the particulate contamination, of silicon during the manufacture of microprocessors [5]. These processes take place in large quantities of ultrapure water, and the reclamation/reuse/recycle of this water could be the source of “biochip” manufacturing raw materials. Other extremophilic microorganisms are most likely to be trapped inside the semiconductor crystal, in a similar manner. For discriminating between the nucleation by microbial exudates or by microbial surface directly, further work has to be performed [1]. Since the morphologically best and potentially most active bio-crystals yet produced, as illustrated in Figure 1, were formed in a fab operating under the Year 2000 conditions, it is important for future improvements to more carefully examine those conditions (which may have changed since then) to inform future deliberate manufacturing attempts. These issues are described below.

Organic impurities, and especially bacteria, have been among the most difficult contaminants to control. One bacterium per 100 mL of water was the recommended water quality limit (for maximum-allowable bacterial-concentration) for water used in processing 4 MB (megabyte) Dynamic Random Access Memory (DRAM) devices [6]. As memory densities increased, this already-stringent requirement became morestrict. Fabs generally monitored only planktonic bacteria (bacteria that are freely suspended in the water) in their UPW systems using nutrient-media methods (e.g., growing the bacteria on R2A or TSA medium) [7]. Using these techniques, up to a week passed between sampling time and detection of the cells, and semiconductor products could be lost by the time a problem was detected. The research already described above showed that the vast majority of the extremophilic bacteria surviving in ultra-pure water systems comprised sessile organisms (bacteria that attach to wetted surfaces), and were not detected with planktonic-bacteria samples. These sessile bacteria, and their associated metabolic residue (exopolymers, waste products), can detach from the wetted surfaces of the water system and secondarily be deposited onto the wetted surfaces of the manufactured product (wafers, connectors), causing defects. Perhaps modern sensors based on this effect can turn this adversity into a virtue by converting the bacterial contamination process into an affirmative enabling technology for advanced devices? These are tasks identified to advance toward that final biosensor production goal, emphasizing those conditions prevailing prior to the Year 2000 when the first bio-crystals were discovered:

(*a*) *Identification of Extremophilic Bacteria and Factors that Influence Bacterial-Nucleation and Growth of Semiconductor or Semiconductor Oxide Crystals*

It is not likely that all microorganisms will be amenable to survival in similar processes, or to incorporation in secondarily nucleated crystals. Process chemicals such as ionic and non-ionic surfactants were used to improve the wettability, reduce the roughness, and reduce the particulate contamination of silicon during the manufacture of microprocessors [5]. They were also used to control the rate of silicon-etching alkaline cleaning solutions, where quaternary ammonium hydroxides are also present [8]. These processes took place in large quantities of ultrapure water, and the reclamation/reuse/recycle of this water could be the source of “biochip” manufacturing raw materials.

Surfactants that were used in microelectronics manufacturing included alkyl phenoxy polyethylene oxide alcohol (e.g., Triton X), fluorinated alkyl sulfonates, and betaines. There is evidence in the older literature that these compounds can be utilized as carbon and energy sources by some bacteria isolated from the environment. This can occur under either anaerobic or aerobic conditions [9–11]. Although many of the hundreds of surfactants present in household and industrial wastes have been shown to be biodegradable (this includes fluorinated surfactants [11]), there is little information about the type of bacteria responsible for the degradation, or the bacterial biochemistry and catabolic genetics involved; exceptions include some sulfonated surfactants [12–15] and alcohol ethoxylate surfactants [16], where the biochemistry of desulfonation and ether-bond cleavage are well established in certain strains of bacteria.

(*b*) *Identify Key Bacteria and the Process Chemicals that Serve as Bacterial Nutrients and Secondary Semiconductor Growth Promoters*

The ultimate goal is to optimize any process chemicals and processes that can be made to control the bacterial growth and micro-bio-semiconductor integration. Projects completed in Northern Ireland have previously included the detailed biochemical and molecular analysis of haloalkane and polycyclic aromatic hydrocarbon degradation [17–22]. One can further characterize rinse/recycle waters obtained from actual fabrication facilities, and isolate bacteria that utilize the process-chemical impurities in these waters to identify key strains that degrade the rinse-water constituents, and prepare cultures of isolates [23] to investigate rates of bacterial growth and process-chemical degradation. The key strains can then be used in biosurface experiments such as those described in the Experimental section of this review, with respect to documenting practical growth conditions and modeling. The identification of additional key bacteria also will provide needed insight for the development of facile bacterial-semiconductor integration.

(*c*) *Detection of Micro-Bio-Deposition and Mineralization*

A production line is often stopped if the numbers of planktonic bacteria exceed acceptable levels. Because the bacteria are first detected by the slow growth of bacterial colonies on nutrient media, the order to stop production is usually not given until days after the contamination actually erupts; thus allowing the production of contaminated product, and generating expensive losses, during the time between bacterial sampling and detection. The lack of a timely contamination-warning method also prevents action that could be taken to avert a costly shut-down. A real-time in-situ monitoring technique, such as that described in the early part of the Experimental section above, would allow more-prompt corrective action to be taken in response to micro-biocontamination problems. Fluorometric-based and improved planktonic-bacteria assays could also offer faster and easier ways to detect bacteria than can be obtained with current sampling protocols.

(*d*) *Monitor Real-Time Micro-Biocontamination*

Real-time, *in-situ* methods are well advanced, as described above, for measuring the accumulation of micro-biocontaminants in commercial processes. Departing from previous industrial sampling practices, these methods have the goal to measure the accretion of sessile bacteria (those attached to wetted surfaces), rather than the concentrations of planktonic bacteria (those freely suspended in the water). Sessile-bacteria measurements will alone yield a direct indication of micro-biocontamination at the location of measurement, since concentrations of planktonic bacteria are essentially functions of the dynamics of sessile populations [24–26]. Although some prior scientific literature contains descriptions [27–31] of biofilm-measurement techniques (e.g., Quartz Crystal Microbalance, and ATR/FTIR Attenuated Total Reflectance/Fourier Transforming Infrared Spectroscopy) that have been used in laboratory flow-cell arrangements, papers describing measurements made in actual industrial environments are rare [27].

(*e*) *Develop New Assays*

A method is desired that will provide a means of quantifying the bacteria on test-coupon or swab samples retrieved from a water system. Unlike planktonic-sampling methods, test-coupon and swab samples should have sufficiently large numbers of bacteria, and not require filtration, concentration, or amplification; the sessile bacteria greatly out-number the planktonic bacteria in UPW systems [24,32].

One early study estimated the ratio of the number of sessile:planktonic bacteria, in a section of pipe distributing high-purity water, to be at least 10^6^:1 [33]. One of the methods of sessile-bacteria measurement that has already been considered uses a commercially available chemical probe, PicoGreen® (Molecular Probes, Eugene, OR, USA) that fluoresces after reacting with chromosomal DNA. When PicoGreen® is used with a standard lab fluorometer (such as the TD-700; Turner Designs, Sunnyvale, CA, USA) a detection limit of 25 pg of DNA per mL of solution can be achieved. This corresponds to the quantity of DNA contained in about 3000 bacteria per mL of solution, assuming the genome of each bacterium in almost all of the bacteria isolated from UPW environments are Gram-negative rods [24]; about 4 × 10^6^ base-pairs [34,35] and a mass of about 9 fg of chromosomal DNA [34]. This resolution would be satisfactory for the analysis of coupons, as the germanum prisms noted above have surface areas of 10 cm^2^, and the bacterial densities on the wetted surfaces (sides of the pipes or vessels of high-purity water systems) are expected to be from 10^4^ to 10^6^ bacteria per cm^2^ [33].

Another method of sessile-bacteria detection to be considered involves adding a fluorogenic substrate (L-Leucine-4-methyl-7-coumarinylamide hydrochloride) to an aqueous sample that contains bacteria. This substrate is hydrolyzed by a particular type of enzyme (leucine aminopeptidase) that is present in most of the bacterial species that occupy aquatic habitats [36]. Upon hydrolysis, a fluorescent compound (7-amino-4-methylcoumarin) is produced. If a saturation dosage of substrate is applied to samples, and if steps are taken to minimize and control mass-transfer effects, bacterial quantities can be estimated by recording the initial rate at which the fluorescent compound is produced. The enzyme-assay method should have greater bacterial-detection capability than the probe method; for the detection of small quantities (<10^3^ bacteria/mL of solution) one should only need to extend the time of the enzyme-substrate reaction, so that a sufficient amount of fluorescent product can be made. As with the probe assay, the enzyme-substrate assay would be similarly inexpensive and simple.

(*f*) *Improve Planktonic-Bacteria Assays*

In industry, aqueous solutions that contain surfactants are often used to clean the fouled surfaces of RO and NF membranes, employed in UPW systems. These treatments were intended for limited use, because excessive contact of RO and NF membrane surfaces with surfactants (especially ionic surfactants) is known to cause considerable reduction in water permeation flux [37]. The permeation and separation behaviors of RO and NF membranes that are continually exposed to an aqueous surfactant-solution are unknown. The ultrafiltration of micellar surfactant-solutions has been studied [38] for cases where the surfactant adsorbs to the membrane surface, and should be extended to bacteria-containing systems.

(*g*) *Select Appropriate Advanced Oxidation Processes*

Current oxidation-methods are not adequate for destroying many organics, so new and advanced oxidation processes (AOP's) are being continually developed. A very promising method is the use of catalytic oxidation, which can mineralize organics. An AOP is based on free-radical, chain-reaction chemistry, in which high energy oxidants are catalytically decomposed to form free radicals such as hydroxyl radicals (OH ·). Frequently employed systems use photons generated by ultraviolet (UV) radiation having a wavelength of 254 nm or 185 nm, as in some of the studies mentioned above. Ozone (O_3_) and hydrogen peroxide (H_2_O_2_) are the most prominent oxidants. In some processes, O_3_ may be combined with H_2_O_2_, either in the presence or absence of UV light, to form OH· [39–41]. In practice, the UV-photolytic generation of OH· is greater with O_3_ than with H_2_O_2_, because of hydrogen peroxide's exceptionally low molar extinction coefficient [42]. An advantage of using H_2_O_2_, rather than O_3_, is the ease of handling. Hydrogen peroxide is produced in 35 to 50% aqueous concentrations and requires minimal capital equipment for transfer to the reactor. Another advantage of using H_2_O_2_ is that it is infinitely soluble in water. Therefore, it does not present a mass transfer problem, as does the transfer of ozone gas to water.

Special emphasis could be placed on heterogeneous photocatalysis as a relatively-new AOP in which a semiconductor (usually titanium dioxide [TiO_2_]) is immersed in water, and illuminated with UV light (wavelength <390 nm). In an aqueous environment, this radiation excites the generation of electrons and holes by the TiO_2_, producing superoxide radicals and hydroxyl radicals [43]. Not only can this AOP completely mineralize various organic pollutants [44], but this technology is also effective for the destruction of microbes [43,45–47]. Sierka and Hendricks [48] demonstrated that the generation of free radicals can be achieved with the addition of ozone to a TiO_2_-photocatalytic system. For the UPW photocatalytic/photolytic process it is possible to design a reactor element that contains a TiO_2_-coated-glass mesh or grid to which UV light can be optically coupled (e.g., by optical fibers, lenses, or mirrors). This would efficiently use and concentrate the light energy, and it would evenly distribute the free-radical oxidants throughout the cross-sectional area of the flow stream.

Another treatment that might be used to generate high concentrations of OH· involves the ultrasonically-induced decomposition of O_3_, H_2_O_2_, and H_2_O. Sierka and Amy [49] suggested the possibility of increasing the production of free radicals by an ultrasound-based effect on ozone. Evidence for free radical formation in water by ultrasound was reported by Mankino [50]. The generation of OH· is related to three major effects caused by the ultrasonification of water, namely: (1) sonic pressures, which create regions of compression and rarefaction in the water; (2) cavitation, which is caused by a three-step process that consists of nucleation, growth, and collapse of vapor-filled bubbles, leading to high pressures (75,000 psi) and temperatures (13,000 °F) of the collapsing bubble; and, (3) microstreaming, by which intense vibrational energy is admitted to a small water volume with little heating. This might be the final driver of an efficient microchip manufacturing process.

## 4. Conclusions/Outlook

It is surprising that a relatively common disease-causing microbe [51] has appeared under extremely organic-free ultrapure water conditions, but it is clear from the discoveries reported that living microbes can exist within ultrapure water systems, and may—based on that observation—be selectively used to corrode/crystallize around themselves semiconductor substances that could serve novel sensor needs. Remaining unknown is the influence of the myriad of compounds and processes within a fabrication facility that influenced the apparent “perfection” of this process, reviewed here from the then-extant literature, and could be selectively chosen for use in off-line commercial fabrication systems that can be turned toward manufacturing value. Progress that has been made over the past decade has, so far, shown the sensitivity of the process only to water purity, flow rate, and time of exposure. The field is more than ready for additional experimental efforts.

## Figures and Tables

**Table 1.** Flow rate and subsequent Shear Rate of Ultra-Pure Water.

	**Flow Rate (mL/min)**	**Shear Rate (s**^−^**^1^)**
Ultra-Pure Water	1 mL/min	3.2
	2.5 mL/min	7.69
	4 mL/min	12.64

**Table 2.** Experimental flow condition during flow of Ultra-Pure Water.

**Flow rate (1mL/min)**	**1 mL/min**	**2.5 mL/min**	**4 mL/min**
Days	After 4	After 4	After 4 Days
	After 7	After 7	
	After 9	After 9	

**Table 3.** Only Ultra-Pure Water at different pH (=5.2).

**Flow Rate**	**1 mL/min**	**2.5 mL/min**
Days	After 4	After 4
	After 7	After 7
